# Health Aspects of the Pre-Departure Phase of Migration

**DOI:** 10.1371/journal.pmed.1001035

**Published:** 2011-05-24

**Authors:** Brian D. Gushulak, Douglas W. MacPherson

**Affiliations:** 1 Migration Health Consultants, Inc., Cheltenham, Ontario, Canada; Qualicum Beach, British Columbia, Canada; 2 Department of Pathology and Molecular Medicine, Faculty of Health Sciences, McMaster University, Hamilton, Ontario, Canada

## Abstract

In the second article in a six-part *PLoS Medicine* series on Migration & Health, Brian Gushulak and Douglas MacPherson discuss the pre-departure phase of migration and the specific health risks and policy needs associated with this phase.

**Summary Points**

- The local conditions and environment at the place of origin of migrants influences health during all phases of the migration process.
- Pre-departure health characteristics are important drivers in health activities directed at migrants, such as immigration medical screening.
- Some pre-departure health elements continue to affect migrant populations long after their arrival at their destination.
- Improved understanding and management of pre-departure health determinants will support the development and delivery of migrant-relevant health services.

**This is one article in a six-part** ***PLoS Medicine*** **series on Migration & Health.**

## Introduction

The flow of populations within and across international boundaries is an important element in today's globalized world. Recent estimates of migration patterns place the combined numbers of international migrants and internal migrants at nearly a billion people [1]. Although migrant populations are extremely diverse, the processes of migration include certain characteristics shared by all migrants. All migrants have a place of origin. Experiences and exposures at a place of origin can influence migrants' health throughout the process of mobility [2], which may include transition, temporary residence, and arrival at a destination. After arrival or settlement, some migrant cohorts may experience ongoing or return migrations that can also have health consequences. [3] As indicated in Table 1, rates of departure from origin countries are markedly different between global areas and countries, with rates in Europe, Latin America, and Oceania more than double those of Africa, Asia, and North America [4]. It is important to note, however, that even low rates of departure from highly populated countries of origin can produce large health impacts at destinations.

**Table 1:** General emigration rates for 89 destination countries (modified from reference [4]).

Origin	Emigration Rate of Population Aged 15 and Older (%)
Global	2.38
High income	3.05
Upper middle income	4.41
Lower middle income	2.02
Low income	1.73
Africa	2.00
Asia	1.16
Europe	5.80
Latin America	5.70
North America	0.92
Oceania	4.52

**Figure 1:**
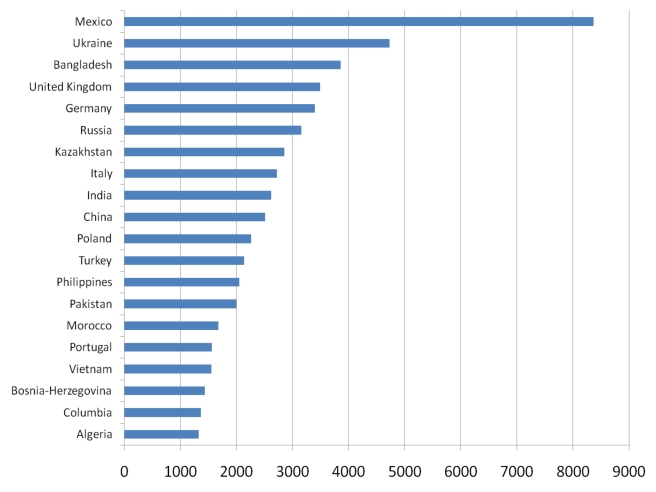
Origin of emigrants (15 years and older) residing in 89 destination countries in 2000. Modified from reference [4].

In general, most migrants move to destination countries in the same region. A recent Organisation for Economic Co-operation and Development (OECD) analysis involving 89 reception countries [4] noted intra-regional emigration flows of 85% in Africa, 75% in Asia, 62% in Latin America, and 60% in Europe. Two other smaller patterns are observed, however, in situations where historical links (e.g., Latin America–Europe) to other regions exist, or where long-standing immigration settlement policies (e.g., Australia, Canada, United States) affect origin and destination dynamics. Global studies of emigration reveal a relative gender balance in aggregate migrant population. However, there are large differences at the continental, regional, and country level (see Figure 1). The same OECD database study indicates that women make up greater proportions of North American and European migrants, while they represent lower proportions of African migrants, especially those from North Africa.

## Migrant Health in the Context of the Pre-Migration Phase

The observation that one's origin, in terms of physical location and the determinants of health (socioeconomics, genetics and biology, behaviour, and environment), influences one's current and future response to events is widely appreciated across the spectrum of social and physical sciences [5]. In the context of migration and population mobility, the pre-departure phase can be considered as the beginning of the migration process and as such affects the rest of the migratory journey. The health characteristics of pre-departure migrant populations can be very diverse, reflecting disparities in the determinants of health at both individual and societal levels. The interaction between those pre-existing determinants of health and the forces that create migration affect many health outcomes in migrants.

Population mobility and migration are the result of a combination of “push” and “pull” factors that are inter-related and often mutually dependent. Descriptions of these factors and examples are provided in Table 2. For example, poverty and under-employment may “push” people to leave their place of residence to a destination that is at least perceived to offer wealth and job opportunities that “pull” migrants [6]. Similarly, environmental forces such as those resulting from natural disasters may generate “push” factors that force people to seek new homes. The combination of environmental and socioeconomic “push” factors such as floods or drought in areas of pre-existing areas of poverty can generate new directions in population flow. Those new patterns can be associated with different health impacts than pre-disaster migration movements. A recent example is provided by migration from Haiti where cholera, a post-2010 earthquake issue, may affect the health of potential migrants [7].

**Table 2:** Examples of determinants of health and mobility impacts.

Type of Influence	Example	Region Affected	Population Affected
Economic	Poverty / unemployment / underdevelopment	Less developed nations / rural areas (both international and internal migration)	Economic migrants / migrant workers / undocumented migrants / adopted children / trafficked migrants
Social	Education / services / opportunity	Global	Immigrants / international students / migrant workers / adopted children
Environmental	Natural disasters• chronic (i.e., desertification, post-volcanic temperature changes)• acute (i.e., earthquake, typhoon, flooding)Man-made disasters (both chronic and acute)• toxic/chemical exposure• radiation release/exposure	Less developed nations (international migration) / global (internal migration)	Refugees / migrant workers / undocumented migrants / adopted children
Conflict	War / insurrection / revolution	Global (internal and international migration)	Refugees / asylum seekers / undocumented migrants / adopted childrenMilitary/armed forces (both volunteer and/or conscripted and coerced)
Political	Repression / discrimination	Global	Refugees / asylum seekers / undocumented migrants

## Factors Generating Migration Flows

These push and pull pressures are unequally distributed across pre-departure migrant populations, and together they both influence and affect migrant demography. An illustration is provided by comparing rural to urban migration and international migration from the same area. Rural to urban internal migration often represents the movement of workers, either with or without their families, from less affluent areas to metropolitan centers where jobs are perceived to be more plentiful. This broad pattern of migration has its own set of health issues and examples have been observed in several locations including child health in Africa, where death in those younger than 5 years old was greater for children of rural–urban migrants [8]. Other examples include the acquisition of less healthy determinants associated with urban living related to diet, activity, body weight, and access to preventive health services. Studies have noted increases in body mass index and diabetes in rural–urban migrants in India [9], increased cardiovascular risk factors in urban migrants in Latin America [10], and reduced rates of immunization in children of urban migrants [11].

Reflecting the diversity of population mobility, not all the effects of rural–urban migration are negative. For example, some studies have noted reduced rates of cardiovascular disease in non-migrant rural populations in South America compared to those in urban migrants in the same country [12]. At the same time, more affluent and educated cohorts from countries experiencing rural–urban migration move internationally as tourists, students, business travellers, and/or as permanent immigrants. For example, by the end of 2009, government estimates of rural migrant workers in China stood at 149 million [13]. Simultaneously, China is a major source of permanent immigrants and international students for nations such as Australia, Canada, the US, and Europe. Pre-migration health, social, and economic conditions will differ between each group even though they originate in the same country. The outcomes of the interaction between these push and pull factors can be important. Wealthy nations with relatively small domestic populations can provide work and residence to large numbers of migrant workers. Health characteristics and outcomes in migrants may differ from those of the domestic host population and may also impact the future health outcomes of the receiving nation.

Some migrants may be more vulnerable to adverse health outcomes. Refugees and displaced populations represent specific populations at risk [14]. In addition to their “normal” pre-migration state, their health status may have been compromised by lack of access to adequate nutrition, health care, public health programs such as routine childhood immunization, or housing during the process that made them refugees [15]. Those who are fleeing conflict may also be subject to violence and trauma, or abuse. The health characteristics of some vulnerable populations, such as permanently settled refugees, are often studied by receiving nations [16]. However, the permanently resettled (112,400 in 2009) represents only a fraction of global refugee and displaced populations (43,000,000 in 2009) [17]. The poor and those acutely displaced by catastrophe or conflict often have less access to, or support for, organized methods of migration and may turn to irregular patterns of population mobility such as illegal or illicit migration, or human smuggling and/or trafficking. By its nature irregular migration is very difficult to quantify, but crude estimates attest to its current and growing importance [18]. Attempting to enter other nations by irregular or illicit means is frequently associated with adverse health outcomes that include injury, exposure to harsh environments, violence, and death [19].

## Health Outcomes in Relation to Pre-Departure Determinants

Pre-departure health status affects both individual and population health outcomes [20]. As described in Table 3, the magnitude of those influences is dependent upon the diversity (differences) and/or disparity (differences with a disadvantage) in the determinants of health and their outcomes between their new destination and those at the migrants' origin. People moving between regions of high endemicity for a disease can carry that epidemiology to low incidence, migrant-receiving nations [21]. Pre-departure differences in chronic disease epidemiology between migrant origin and destination locations can have long-term effects [22]. Over time and with sustained migration from high prevalence to low prevalence areas, migrants can come to represent specific disease risk groups in destination countries [23] for non-prevalent conditions such as tuberculosis [24], hepatitis B [25], strongliodaiasis [26], malaria [27], cystercercosis [28], South American trypanosomiasis [29], diabetes [30], renal failure [31], cardiovascular disease [32], and certain malignancies [33], among others.

**Table 3:** Pre-movement factors that influence health (modified from reference [71]).

Factor or Condition	Individual and Population Outcome
Incidence and prevalence of infectious diseases, e.g., tuberculosis, hepatitis B	Transmission of or acquisition of disease during journey or on arrival
Incidence and prevalence of non-infectious disease/illness, e.g., pregnancy, hypertension, diabetes	Introduction of individual/population with different health characteristics/needs into the receiving health care system
Social factors (education/housing/poverty), e.g., behavioral effects on health including nutrition and diet; access to and use of care; management of existing illnesses; violence (interpersonal and/or domestic); risk-taking (tobacco/substance abuse)	Baseline levels of health status that can increase the risk of illness/disease during travel, and affect access to health services on arrival
Environmental factors (geographic, weather, toxic, political), e.g., post-traumatic stress disorder, abuse and torture	Background level of nutrients, toxins, violence, trauma (physical/psychosocial), and natural events (extreme temperatures, storms, fires, earthquakes)
Factors related to pre-departure migrant status, e.g., refugee, irregular migrant, migrant worker, immigrant	Availability, accessibility, and affordability of existing health and social care services (limited access to insurance/care; capacity to provide services for trauma/torture; occupational health needs)
Cultural/experiential factors, e.g., differential in health services utilization and expectations	Expectations and utilization of health services/concepts of disease and ill health. The institutional and non-institutional capacity to provide for and respond to needs for health promotion, prevention, and intervention in diverse populations.

Not all of the health concerns in migrants that are the consequences of geographically disparate disease epidemiology are related to infectious diseases. Health outcomes in migrants also include biological and inherited elements as well as those associated with ethnicity and social and cultural practices, as reflected in the selection of marriage partners [34]. Some genetic conditions, such as the hemoglobinopathies more common in the Levant and other areas [35], have post-immigration implications in locations where these genetic features had not evolved and were not normally distributed [36]. The introduction of sickle cell disease into the Americas [37] or the differences in malignancy incidence reflected in some migrant populations embedded in host environments [38] are examples of these impacts. Another example is provided by studies on the international movement of *Helicobacter pylori* [39], which has post-infection, chronic consequences, including malignancy.

Historically, there has been a tendency to consider only the adverse health risks related to migration, focusing on disease risks in migrant populations that were greater than the host population. It is important to note, however, that the consequential health outcomes for both the migrant and host population may be positive, neutral, or negative.

In several migration-receiving nations, cohorts of new arrivals often display health characteristics that are better than that those of similar cohorts of the domestic population. These observations are frequently related to lifestyle choices or chronic diseases (e.g., dietary choices, physical fitness, smoking, substance abuse) but extend to other situations (e.g., use of health services, fecundity and pregnancy outcomes). Described as the “healthy immigrant effect” [40], examples of this type are important in defining migrant factors that impact health outcomes.

## Health and Health Service System Implications of the Pre-Departure Phase

Historically, the pre-departure influences affecting the health of migrants were approached in terms of the potential risks migrants were believed to pose to the domestic host population. Attempts to control the admission of epidemic diseases grew to include the medical screening of arriving migrants [41]. Practiced by nations with organized immigration selection programs, medical screening may be an element of a formal regulated process used to determine the eligibility of entry on health grounds [42]. Additional or supplemental screening is often recommended for clinical or public health benefit [43]. Screening is also a frequent component of organized migrant labor or temporary workers programs in Asia [44] and the Middle East [45].

The nature, purpose, and type of migrant medical screening for exclusion varies by nation from none at all to very detailed, proscriptive programs [46]. Those screening immigrant programs that do exist commonly include testing for communicable diseases of public health significance (e.g., tuberculosis and a small number of other infectious diseases); chronic diseases that may impact health or social services (e.g., cancer, heart disease, mental disability); or medical conditions deemed to be a social risk factor (substance abuse, mental disease). Screening of migrants may be enhanced or introduced in situations of international public health concern such as was observed in SARS [47], human infection with avian influenza, and the H1N1 (2009) influenza pandemic [48]. Screening for migrant labourers may include aspects of fitness for work.

Some nations with universal health insurance systems, such as Canada [49] and Australia [50], apply immigration screening to prevent the admission of some complex or costly diseases that could adversely affect the domestic supply of limited health services. Nations that screen migrants in terms of disease cost or service demand often waive these requirements for refugee or humanitarian migrant populations.

More recently, expanding the concept of immigration medical screening is being considered in terms of screening not for exclusion on health grounds [51], but as a tool to assess the public health fitness of the newly arriving migrant [52] and perhaps facilitate integration into the health systems at the migrants' destination. While these approaches are still being developed, some steps in this regard are being undertaken. Immunization against vaccine-preventable diseases may be required by some migrant-receiving nations [53], and special populations at risk such as refugees or adopted children may receive additional attention. Immigration screening in this context has the potential to become an integral component of public health promotion and prevention in migration receiving countries.

An additional pre-departure health element that exerts influence after a migrant's arrival is the approach to the use of health services. Models of health care delivery differ across the globe. Examples include ayurvedic and traditional Chinese medicine used by billions of individuals, which differs from Western allopathic medicine [54]. Migrants arriving from backgrounds where different medical models of care are used may use host country medical services differently [55],[56]. Those arriving from fee-for-service environments may be unaware or unfamiliar with the provision of nationally insured services, for example [57]. At the same time, fear of potential consequences, migrants' perceptions and attitudes, and provider competency may defer or delay migrants' use of medical services [58].

## Policy Challenges Posed by Pre-Departure Health Factors

Migration health policies, when they exist, are frequently based on traditional considerations of immigration/emigration. Those frameworks often categorize mobile populations of increasingly diverse origin into a limited number of administratively determined immigrant categories. Health concerns in mobile populations have often been addressed in terms of traditional migrant classification (refugee, immigrant, temporary worker, visitor, etc.). While those categories may reflect historical migration flows, they are often not representative of modern migrant diversity or disparity, nor may they reflect the current reality of health differences relevant to receiving nations. An example is provided by the demographic, experiential, and personal differences present in current refugee populations. Depending on location and national practice, a wealthy, educated political refugee originating in a developed metropolitan area who filed an asylum claim versus an economically and educationally deprived laborer forced from his or her home into a refugee camp by conflict, could be administratively classified identically. Yet, their health status and needs may be significantly different.

Recommendations to consider health policies and programs for migrants in terms of the country of origin as a reference point rather than immigrant classification began in the 1980s [59]. More recently the need to expand the scope of migrant health policies to include additional parameters beyond the traditional administrative labels is also becoming better appreciated [60]. This increased appreciation of the health implications of modern migration includes national, bilateral and multilateral approaches to managing health disparities in some migrant populations. Some European nations that receive large numbers of migrants from less developed areas, including Spain and Italy, have extended municipal or national health insurance coverage for migrants [61]. In Canada, the federal government offers health coverage for refugees and refugee claimants until they qualify for provincial health insurance [62].

The repetitive, cyclic flow of migrants, such as migrant labor or migrants visiting friends and relatives in their place of origin, can create specific health challenges that exceed the capacities of traditional programs developed for uni-directional migration. Nations sharing common borders frequently crossed by migrants are developing joint projects to manage health issues in mobile populations. Examples include shared programs along the US–Mexican border that involve common health information systems and shared treatment and monitoring systems [63]. Other examples include guidelines for the assessment and management of health conditions in migrant travellers at specific risk, such at those who visit friends and relatives [64]. Globally and regionally integrated public health surveillance and monitoring of pre-departure health characteristics can provide early recognition of disease or illness in migrants and other mobile populations. Examples include surveillance and monitoring systems for tropical infections, such as TropNetEurop [65], and for travel-associated illnesses, such as GeoSentinel [66]. Through these multi-site systems, providers and laboratories report imported or travel-related diseases in an aggregated format that allows for the early identification and quantification of risks in mobile populations, including migrants. This information is used to support disease prevention activities and management activities and programs.

The cumulative implications of the pre-departure health status of migrants ultimately extend to the delivery of patient care at the destination. Cultural competency and the ability to deal with diversity are increasingly important aspects of health care in migrant-receiving locations [67]. Migrant-receiving destinations are increasingly faced with the need for linguistic and cultural services to reduce barriers to care posed by language and different cultural norms. These needs extend to the level of the clinical caregiver who, in an increasingly globalized world, requires greater awareness of pre-departure factors for migrant populations in order to accommodate specific migrant needs [68].

## Conclusions

The determinants of health present during the pre-departure phase of migration are crucially important factors affecting the existing and future health outcomes of migrants and host populations. The effects of these factors extend throughout the remaining phases of the migratory process and apply at both the individual and population level. Appreciating and dealing with these issues at operational and policy levels requires global focus, rapid and flexible response to change, and current information on the composition and nature of the migrants themselves as opposed to traditional administrative migrant-classification- or disease-based paradigms. Increasingly, the challenges of dealing with migrant health are being addressed through collaborating centers of reference and experience [69],[70]. Bringing together multidisciplinary sectors that include providers, migrant communities, and educational institutions, these centers allow for the effective preparation of migrant-focused policies, programs, and services using shared knowledge, research, and resources. Collaboration of this type reduces duplication of activities, allows for the expedient extension of best practices, and supports comparative research.
